# The Impact of COVID-19 Vaccination on Symptoms of Anxiety and Depression before and after COVID-19 Vaccines Were Universally Available for Adults in the United States

**DOI:** 10.1155/2024/9682710

**Published:** 2024-05-07

**Authors:** Angela M. Parcesepe, Denis Nash, Jenny Shen, Sarah G. Kulkarni, Rebecca Zimba, William You, Amanda Berry, Rachael Piltch-Loeb, Sasha A. Fleary, Eva Stanton, Christian Grov, McKaylee M. Robertson

**Affiliations:** ^1^Department of Maternal and Child Health, Gillings School of Public Health, University of North Carolina, 427 Rosenau Hall, CB #7445, Chapel Hill, NC 27599-7445, USA; ^2^Carolina Population Center, University of North Carolina at Chapel Hill, Chapel Hill, NC, USA; ^3^Institute for Implementation Science in Population Health (ISPH), City University of New York (CUNY), New York, NY, USA; ^4^Department of Epidemiology and Biostatistics, Graduate School of Public Health and Health Policy, City University of New York (CUNY), New York, NY, USA; ^5^Emergency Preparedness Research Evaluation and Practice (EPREP) Program, Harvard T.H. Chan School of Public Health, 90 Smith St, Boston, MA 02120, USA; ^6^Department of Community Health and Social Sciences, Graduate School of Public Health and Health Policy, City University of New York (CUNY), 55 W 125th St, New York, NY 10027, USA

## Abstract

Our objective was to examine the influence of COVID-19 vaccination on recent (i.e., past month) moderate or severe symptoms of anxiety (GAD‐7 ≥ 10) or depression (PHQ‐8 ≥ 10) before and after the COVID-19 vaccine became universally available for adults in the U.S. Participants belonged to the Communities, Households, and SARS-CoV-2 Epidemiology Cohort (CHASING COVID), a national longitudinal study. Our analytic population included 4,832 participants who reported vaccination status from December 2020 to December 2021 with follow-up outcomes assessed through March 2022. We emulated a hypothetical randomized experiment, a target trial, to estimate the effect of COVID-19 vaccination on symptoms of anxiety or depression. Before vaccines were universally available, participants who were vaccinated versus not had significantly lower adjusted odds of symptoms of moderate or severe anxiety (aOR: 0.79; 95% CI: 0.70-0.89). In the universal vaccine era, vaccination was associated with marginally higher adjusted odds of symptoms of moderate or severe anxiety (aOR: 1.23; 95% CI: 1.00-1.50). Vaccination did not influence subsequent moderate or severe depressive symptoms in the preuniversal vaccine era (aOR: 0.92; 95% CI: 0.82-1.03) or universal vaccine era (aOR: 1.11; 95% CI: 0.91-1.36). Research into the longitudinal relationship between COVID-19 vaccination and symptoms of depression and anxiety is warranted, with a focus on advancing understanding of potential mediators on the pathway between vaccination and mental health as well as modifiable factors, such as vaccine hesitancy or vaccine beliefs, that may help identify populations for whom vaccination may be particularly beneficial to their mental health.

## 1. Introduction

The COVID-19 pandemic and associated mitigation strategies and consequences have had significant mental health impacts [1–3]. Symptoms of anxiety, depression, and stress have increased among the general population and among vulnerable groups [4, 5]. In addition, the prevalence of individuals who needed but did not receive mental healthcare has increased [6]. A number of factors have contributed to the increased mental health burden during the pandemic including social distancing, increased unemployment and financial hardship, increased loneliness and social isolation, fear and anxiety related to COVID-19 exposure and transmission, COVID-19 infection and long COVID-19, loss of loved ones, and disruptions to health services, including primary care and mental healthcare [7–11].

In December 2020, the Food and Drug Administration (FDA) approved the emergency use authorization (EUA) of COVID-19 vaccines developed by Pfizer and Moderna [12]. On April 19, 2021, the Biden administration announced that all people in the United States aged 16 or older were eligible for COVID-19 vaccines [13]. As of January 2023, 79% of U.S. adults have received a complete primary COVID-19 vaccine series and 18% have received an updated bivalent COVID-19 booster dose [14]. A robust evidence base demonstrates the safety and efficacy of these vaccines in preventing severe COVID-19 illness, hospitalization, and death, and more recently, long COVID-19 [15–18].

Little remains known about the relationship between COVID-19 vaccination and mental health, and evidence suggests that holding ambivalent attitudes toward vaccination may be related to poor mental health [19, 20]. For some, the negative effects of the pandemic on mental health may be reduced with vaccination and the possibility of reduced social isolation and increasing population immunity that comes with vaccination [21–23]. This may be particularly relevant for those with greater fear or anxiety related to COVID-19 exposure or illness or for those at greater risk for severe COVID-19 illness [21, 23, 24]. For others, vaccine hesitancy and concerns related to the perceived safety or efficacy of the COVID-19 vaccine may be associated with increased mental distress after vaccination [25]. Some individuals may also remain socially isolated after vaccination, especially elderly individuals or those with comorbidities.

In addition, the relationship between COVID-19 vaccination and mental health may differ among those vaccinated before versus after COVID-19 vaccines became universally available [22]. Individuals vaccinated prior to the universal availability of the COVID-19 vaccine may have been at greater risk of COVID-19 exposure (e.g., through employment) or at greater risk of severe COVID-19 illness or death (e.g., due to underlying comorbidities or older age) compared to individuals vaccinated after the COVID-19 vaccine became universally available. Perceived risk of COVID-19 exposure or severe COVID-19 illness and death may influence the relationship between COVID-19 vaccination and mental health [21]. In addition, the relationship between COVID-19 vaccination and mental health may differ among those with preexisting mental health conditions compared to those without prior mental health conditions [24]. For those with preexisting mental health symptoms, vaccination may have a modest effect on mental health. However, for those with mental health symptoms onset during the COVID-19 pandemic, vaccination may be associated with reduced COVID-related fear and anxiety and improved mental health.

Longitudinal epidemiologic research is essential to assess the role of public health mitigation strategies, such as vaccination, on mental health symptoms. This research can be used to inform, adapt, and target evidence-based mental health interventions for early deployment in future pandemics and other public health emergencies.

### 1.1. Study Objective

Our objective was to examine the influence of COVID-19 vaccination on symptoms of anxiety and depression before and after the COVID-19 vaccine became universally available for adults in the U.S. We also examined the influence of COVID-19 vaccination on symptoms of anxiety and depression separately, among those with symptoms of anxiety and depression prior to vaccination, within a large national cohort of adults.

## 2. Methods

### 2.1. Data Source and Population

The CHASING COVID Cohort Study is a geographically and sociodemographically diverse sample of adults (18 and older) residing in the U.S. or U.S. territories who enrolled in a prospective cohort study during the emergence of the COVID-19 pandemic in the U.S. [26]. Details of cohort recruitment and follow-up have been described elsewhere [26]. We used internet-based strategies to recruit a fully online cohort and recruited study participants from March 28, 2020, to August 21, 2020, via advertisements on various social media platforms (e.g., Facebook), Qualtrics Panel, or via referral (anyone with knowledge of the study was allowed to refer others to the study). Study participants have been prospectively followed, with online assessments occurring approximately every three months from enrollment to the end of analysis follow-up (March 2020-March 2022). The study assessments capture a variety of sociodemographic measures and measures related to COVID-19, including vaccination history. Study materials, including assessments, are available online [27]. Our analytic population included participants who reported vaccination status from December 2020 to December 2021 with follow-up outcomes assessed through March 2022.

### 2.2. Research Ethics Approval

The study protocol was approved by the Institutional Review Board at the City University of New York (CUNY). Informed consent was obtained at study enrollment and updated in March 2022 to account for study extension.

### 2.3. Target Trial Specification

We used CHASING COVID Cohort data to emulate a hypothetical randomized experiment, the target trial. We first outlined the protocol of a target trial to estimate the effect of vaccination on symptoms of anxiety and depression among all adults and among the subgroup of adults with prevalent symptoms of anxiety or depression (see protocol in Supplemental Table 2). Briefly, in the hypothetical target trial, the eligibility criteria were related to eligibility for vaccination, the treatment strategies compared were receipt or no receipt of the COVID-19 vaccine, and participants were followed for short-term (~3 months) symptoms of anxiety or depression.

### 2.4. Target Trial Emulation

Next, we emulated the design and intention to treat (ITT) analysis of the target trial. Our observational “trial” was conceptualized as a sequence of nonrandomized “trials” [28–30], in which participants were allowed to enter into a subsequent trial if they remained unvaccinated during the previous trial (Supplemental Table 3). The study design protocol was applied to each trial.

### 2.5. “Trial” Eligibility, Treatment Assignment, and Follow-Up

We mimicked the design features of an experiment by defining eligibility criteria, treatment assignment (i.e., vaccinated or unvaccinated), and time zero, a person-specific time from which to start follow-up for outcome assessment [29, 31–33]. Participants were eligible if they completed the December 2020 assessment and reported their vaccination status. This was the first assessment to query participants' vaccination status. We conducted an additional 4 nonrandomized “trials” starting at each subsequent assessment through December 2021.

We assigned “treatment” (vaccinated or unvaccinated) and the associated time zero when a participant met “trial” eligibility. To align with ITT principles, we considered time zero to be the month of the first dose for the vaccinated group and the month of assessment completion for the unvaccinated group. We included all eligible assessments when a person was not vaccinated and the first assessment when a person was vaccinated.

### 2.6. Variable Definitions

#### 2.6.1. Outcomes: Symptoms of Anxiety and Depression

We measured symptoms of generalized anxiety using the Generalized Anxiety Disorder 7-item (GAD-7) and depression using the Patient Health Questionnaire 8-item (PHQ-8) at each follow-up assessment. Scores on the PHQ-8 range from 0 to 24. Scores on the GAD-7 range from 0 to 21. We dichotomized these variables as the presence or absence of symptoms of moderate to severe anxiety (GAD-7 score ≥ 10) [34] or depression (PHQ‐8 ≥ 10) [35]. We used the GAD-7 or PHQ-8 score reported in the assessment after the time zero of each trial as the outcome, generally 3 months following vaccine measurement.

#### 2.6.2. Exposure: COVID-19 Vaccination

Receipt of COVID-19 primary vaccine series between December 2020 and December 2021 was assessed by participant self-report. Participants reported vaccination dates and the number of doses of the primary vaccine series received. Participants who reported receiving at least one dose of an EUA-approved COVID-19 vaccine were considered to have started the primary vaccine series. Self-report of COVID-19 vaccine status has high concordance (95.0% [95% CI: 93.9%–95.9%]) with sources such as vaccine registries and electronic health records [36].

#### 2.6.3. Universal Vaccine Era: April 19, 2021

We considered the universal vaccine era to begin on April 19, 2021, when all persons aged 16 or older were eligible to receive a COVID-19 vaccine in the US [37]. We considered participants who reported their first dose on or after April 19, 2021, to have begun their primary vaccine series in the universal era, and participants who reported their first dose before April 19, 2021, to be in the preuniversal era.

#### 2.6.4. Covariates

At study enrollment, we measured sociodemographics (age, gender, race/ethnicity, income, education, and whether children < 18 years lived in the household), the ability to social distance as a measure of potential SARS-CoV-2 exposure, susceptibility to COVID-19 complications, and access to healthcare. The exposure, susceptibility, and access to healthcare measures were derived from a national survey that explored the experience of adults during the 2009-2010 H1N1 influenza pandemic and modified for the COVID-19 pandemic [38–40]. As the measure of potential SARS-CoV-2 exposure, we included built-environment and work-related items that contributed to the ability to social distance: living in an urban area, living in a multiunit dwelling, taking public transportation, being an essential worker, and having the ability to stay home from work or work from home. As a measure of COVID-19 susceptibility, we used conditions or exposures identified by the Centers for Disease Control and Prevention in March 2020 which increased the risk for COVID-19 complications: age, smoking, and underlying chronic conditions. As the measure of healthcare access, we used factors that affect medical care access: no primary care doctor, concerns about the costs of healthcare, concerns about seeing a doctor because of immigration status, or no healthcare coverage/insurance. See the appendix for detailed information.

We also captured time-updated employment status, food insecurity, and housing instability. We used a 2-item screener to assess food insecurity [41, 42] and a single question from the Behavioral Risk Factor Surveillance System to assess housing instability [43].

### 2.7. Statistical Methods

We used multiple imputations for missing outcome data, with 7% of all anxiety or depression scores imputed. The final multiple imputation models for anxiety or depression included age, race/ethnicity, gender, income, education level, employment status, food insecurity, housing instability, having any children < 18 years in the household, access to healthcare, and anxiety (for the anxiety model) or depression (for the depression model) at study enrollment. These variables were identified *a priori* as variables in the exposure-outcome models or as variables that may be highly correlated with missing data, often referred to as auxiliary variables in the multiple imputation literature [44]. We assessed model fit with the Bayesian information criterion (BIC).

To assess the reduction in severity of mental health symptoms following vaccination, we estimated the observational analog of the ITT odds ratio. We used logistic regression models to assess the odds of moderate-to-severe anxiety or depression symptoms by vaccination status, adjusting for confounders measured at or before time zero. The overall estimated measures of effect were created by pooling the emulated nested trials, with each participant in a trial contributing to a person-trial [29, 32]. Since some subjects could be included in more than one “trial” (up to 5), we used a robust variance estimator to account for within-person correlation.

We assessed the effect of vaccination on mental health symptoms in the entire cohort and among two subgroups of people: those with moderate-to-severe symptoms of anxiety and those with moderate-to-severe symptoms of depression as of the *most recent measure at time zero*. In the subgroups, we also ran a linear mixed model to assess the change in GAD-7 or PHQ-8 scores, adjusting for confounders as of time zero with a random intercept for participants to account for repeated measures. Based on an *a priori*-directed acyclic graph (DAG), we identified the observed minimum sufficient adjustment set for estimating the total effect of vaccines' impact on mental health. The final models included age, gender, education, employment, access to healthcare, housing and food insecurity, susceptibility to severe COVID-19 outcome, and anxiety (or depression) status. We assessed the heterogeneity of the ITT effect by vaccine era with the Wald statistic. Because heterogeneity was present, we present our findings separately by vaccine era (preuniversal vs. universal availability).

## 3. Results

### 3.1. Creating a Sequence of “Trials”

**Figure 1:**
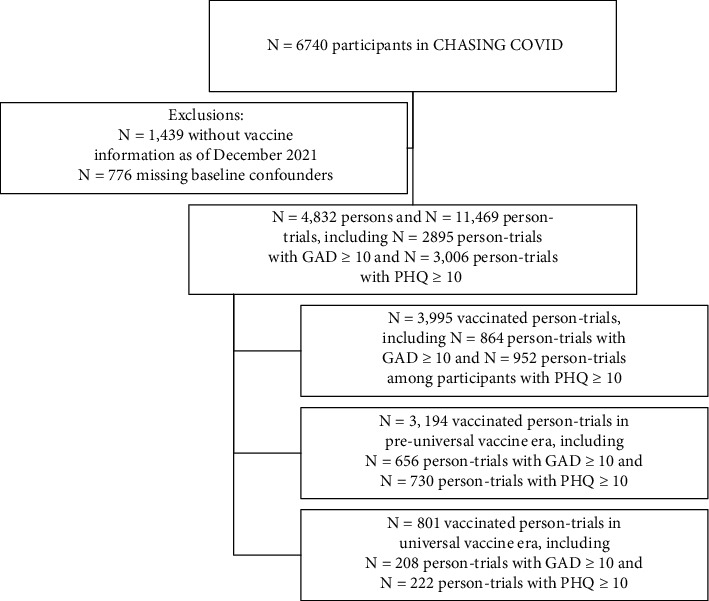
Study flow for inclusion in target trial to assess the impact of COVID-19 vaccine on symptoms of anxiety and depression before and after vaccines were universally available.

Participants were classified into two groups: those who received the COVID-19 vaccine and those who did not receive the vaccine. A total of *N* = 6,740 participants were enrolled in longitudinal follow-up in the cohort, and *N* = 4,832 participants were eligible for the target trial (having documentation of vaccine status and measurement of confounders), and we emulated *N* = 11,482 person-trials (Figure 1), including *N* = 3,987 vaccinated person-trials, with most vaccinated person-trials occurring in the preuniversal era (*N* = 3,191, 80% of vaccinated person-trials).

Table 1 shows sociodemographic characteristics and baseline anxiety and depressive symptoms for participants vaccinated in the preuniversal and universal vaccine eras. Compared to participants who were not vaccinated, a greater proportion of participants who received their first vaccine dose in the preuniversal era were 60 years or older. White, non-Hispanic, wealthier, college-educated, had never experienced unstable housing or food insecurity, and had fewer barriers to healthcare access. Trends were broadly similar in the universal era, in that a greater proportion were vaccinated among those with higher income and a college degree. Sociodemographic characteristics by vaccination status in the pre- and universal-vaccine eras among the subgroups with moderate or severe anxiety or depressive symptoms are reported in Supplemental Table 1.

### 3.2. Relationship between COVID-19 Vaccination and Symptoms of Anxiety and Depression

Before vaccines were universally available, participants who were vaccinated versus not vaccinated had significantly lower adjusted odds of moderate or severe anxiety symptoms (aOR: 0.79; 95% CI: 0.70-0.89) (Table 2). In the universal vaccine era, vaccination was associated with marginally higher adjusted odds of symptoms of moderate or severe anxiety (aOR: 1.23; 95% CI: 1.00-1.50). Vaccination did not influence subsequent moderate or severe depressive symptoms in the preuniversal vaccine era (aOR: 0.92; 95% CI: 0.82-1.03) or universal vaccine era (aOR: 1.11; 95% CI: 0.91-1.36).

### 3.3. Effect of COVID-19 Vaccination on Symptoms of Anxiety among Those with Anxiety at Time Zero

In the preuniversal vaccine era, among those with symptoms of moderate to severe anxiety at time 0, participants who were vaccinated versus those not vaccinated had lower odds of symptoms of moderate to severe anxiety (aOR: 0.67; 95% CI: 0.57-0.80) (Table 3). However, in the universal vaccine era, among those with symptoms of moderate to severe anxiety at time zero, vaccination was not associated with anxiety symptoms postvaccination (aOR: 1.03; 95% CI: 0.78, 1.38). In the preuniversal vaccine era, among those with anxiety symptoms at time 0, those who were vaccinated had a mean GAD-7 score of 1.16 (95% CI: 0.76, 1.55) points lower than those who were not vaccinated. During the universal vaccine era, among those with anxiety symptoms at time 0, the mean GAD-7 score was not meaningfully different between those who were versus those who were not vaccinated.

### 3.4. Effect of COVID-19 Vaccination on Depressive Symptoms among Those with Depression at Time 0

Similar to findings with anxiety symptoms, among those with moderate to severe depressive symptoms at time 0, the relationship between vaccination and moderate to severe depressive symptoms was statistically significant in the preuniversal vaccine era (aOR: 0.83; 95% CI: 0.70, 0.98), but not in the universal vaccine era (aOR: 0.94; 95% CI: 0.71, 1.24). Similarly, among those with depressive symptoms at study enrollment, those who were vaccinated had a mean PHQ-8 score of 0.64 (95% CI: 0.29, 1.00) points lower than those who were not vaccinated. Before vaccines were universally available, among those with depression symptoms at time 0, those who were vaccinated had a mean PHQ-8 score of 0.82 (95% CI: 0.40, 1.23) points lower than those who were not vaccinated. During the universal vaccine era, among those with depressive symptoms at time 0, the mean PHQ-8 score was not meaningfully different between those who were versus those who were not vaccinated.

## 4. Discussion

COVID-19 vaccination was associated with a reduced burden of anxiety symptoms prior to universal vaccine availability among both the entire study population and among those with anxiety symptoms at time zero. COVID-19 vaccination was associated with a reduced burden of depressive symptoms prior to universal vaccine availability among those with depressive symptoms at time zero. However, this relationship did not persist when examined among the entire study population. Our results are among the first to demonstrate a distinction in the effect of COVID-19 vaccination on adults with anxiety symptoms and depressive symptoms and also among the first to focus on the temporal effect of vaccination on mental health in the preuniversal availability vs. universal availability eras. Results highlight the importance of investigating the impact of public health mitigation strategies, such as vaccination, on individuals with anxiety separately from individuals with depression. Further, our results prompt further research to identify potential drivers of this relationship between COVID-19 vaccination and subsequent anxiety symptoms. Our findings suggest the importance of developing tailored public health and risk communication messaging for individuals with anxiety, especially during public health emergencies when the prevalence of anxiety in the general population is likely to be elevated [45–47].

Our findings are consistent with previous research with a nationally representative cohort of U.S. adults that found that COVID-19 vaccination was associated with decreased psychological distress [21]. While Koltai et al. did not stratify their findings by vaccine era (preuniversal vs. universal availability), vaccination in their study was assessed through June 2021. As such, the majority of the time assessed in their study occurred in the preuniversal availability era. It should also be noted that Koltai et al. measured psychological distress using the Patient Health Questionnaire 4 which inquires about symptoms of both anxiety and depression [21]. Our study expanded on these findings by assessing the relationship between COVID-19 vaccination and symptoms of anxiety and depression separately. In adjusted analyses among the entire cohort, COVID-19 vaccination was associated with lower odds of symptoms of moderate to severe anxiety, but not depression in the preuniversal availability era. Additional research is needed to better understand potential pathways between COVID-19 vaccination and subsequent mental health symptoms. Our study supports that such research should examine this relationship separately for anxiety and depression, as the relationship between COVID-19 vaccination and mental health may differ by symptom type.

In the current study, the mental health benefits of COVID-19 vaccination were concentrated among those vaccinated during the preuniversal eligibility era. Previous research found that the relationship between COVID-19 vaccination and reduced psychological distress was partially mediated through reductions in perceived or actual vulnerability to COVID-19 infection, hospitalization, and death [21]. Given vaccine eligibility requirements implemented in the preuniversal eligibility era, it is possible that individuals vaccinated in the preuniversal eligibility era felt greater vulnerability to SARS-CoV-2 infection, severe COVID-19 illness, or death. However, our models adjusted for susceptibility to severe COVID-19 illness or death and work-related exposure.

In the universal eligibility era, there was a marginal increase in vaccine-associated symptoms of anxiety. Additional research is needed to understand potentially modifiable mediators of the relationship between COVID-19 vaccination and anxiety symptoms, including vaccine hesitancy, vaccine beliefs, perceived risk of COVID-19 infection, and perceived risk of severe outcomes if infected, particularly now that the COVID-19 vaccine is universally available. An increase in postvaccination anxiety symptoms may be of concern for future vaccine uptake. An investigation into the extent to which vaccine hesitancy or vaccine perceptions mediate the relationship between COVID-19 vaccination and anxiety symptoms postvaccination warrants examination, particularly as much extant research has focused on the relationship between vaccine hesitancy and mental health prevaccination. A study with individuals in China found that vaccine hesitancy mediated the relationship between gender, education, employment, and mental health after COVID-19 vaccination [25]. In our results, the increase in postvaccination anxiety symptoms may be of concern for future vaccine hesitancy. Further investigation into interventions that support mental health and reduce vaccine hesitancy is warranted.

Among individuals with anxiety or depressive symptoms at time 0, COVID-19 vaccination was associated with lower odds of moderate to severe symptoms of both anxiety and depression in the preuniversal era. This is consistent with research among adults in Jordan which found that anxiety symptoms decreased significantly postvaccination among individuals experiencing mild to severe anxiety before COVID-19 vaccination, with greater reductions among those with moderate or severe anxiety compared to mild anxiety [24]. Similarly, among a national cohort of adults in Japan, COVID-19 vaccination was associated with improved mental health among those who reported psychological distress before vaccination [23]. Additional research is needed to better understand the mental health impact of COVID-19 vaccination among individuals with mental health symptoms prior to vaccination.

Much work remains to increase COVID-19 vaccination among US adults. The mental health benefits of COVID-19 vaccination could be promoted in public health campaigns, particularly for those with preexisting anxiety or depression. The extent to which COVID-19 vaccination contributes to improved mental health should be explored and characterized further. In addition, the relationship between COVID-19 vaccination of children and parental mental health should be explored, particularly as hesitancy to vaccinate children for COVID-19 has been commonly reported, even among parents who were vaccinated for COVID-19 [48].

### 4.1. Limitations

This work has limitations worth noting. First, vaccine uptake was self-reported and may be biased. In addition, the sample, while large and sociodemographically diverse, was not nationally representative of the U.S. population, limiting our ability to generalize our findings to the US adult population. Further, vaccine uptake was measured as at least one dose of an FDA-approved COVID-19 vaccine. While this aligns with ITT protocols, this may underestimate the mental health effect of a complete primary series or subsequent doses of the COVID-19 vaccine. As with all observational studies, unmeasured confounding is a concern (e.g., COVID-19 vaccine misinformation may have contributed to decisions not to vaccinate and to increased anxiety). We restricted our analysis to those with complete information on all measured confounders, which allowed us to adjust for as many confounders as possible but may introduce selection bias. Participants without completely measured confounders were not meaningfully different than those with completely measured confounders (Supplemental Table 2). Finally, we were unable to account for the local roll-out of city- or state-specific vaccine eligibility guidelines.

This study found that COVID-19 vaccination was associated with improved mental health, with particular benefits among those vaccinated in the era prior to the vaccine becoming universally available and for people with mental health symptoms predating vaccination. Research into the longitudinal relationship between COVID-19 vaccination and symptoms of depression and anxiety is warranted, with a focus on advancing understanding of potential mediators or mechanisms of action on the pathway between vaccination and mental health as well as modifiable factors such as vaccine hesitancy and vaccine beliefs that may help identify populations for whom vaccination may be particularly beneficial to their mental health. The extent to which COVID-19 vaccination of children is associated with parental mental health should also be investigated.

## Figures and Tables

**Table 1:** Sociodemographic characteristics by vaccination status in the pre- and universal vaccine eras—CHASING COVID Cohort, USA, December 2020-January 2022.

	Overall, *N* (%)	Preuniversal vaccine era (December 2020-April 18, 2021)		Universal vaccine era (April 19, 2021-January 11, 2022)	
		Not vaccinated, *N* (%)	Vaccinated, *N* (%)	Not vaccinated, *N* (%)	Vaccinated, *N* (%)
	11,469 (100.0)	4,846 (100.0)	3,194 (100.0)	2,628 (100.0)	801 (100.0)
*Age*					
18-49	8,129 (70.9)	3,390 (70.0)	2,006 (62.8)	2,070 (78.8)	663 (82.8)
50-59	1,536 (13.4)	650 (13.4)	479 (15.0)	325 (12.4)	82 (10.2)
60+	1,804 (15.7)	806 (16.6)	709 (22.2)	233 (8.9)	56 (7.0)

*Gender*					
Cisgender male	4,889 (42.6)	2,152 (44.4)	1,502 (47.0)	892 (33.9)	343 (42.8)
Cisgender female	6,296 (54.9)	2,558 (52.8)	1,594 (49.9)	1,706 (64.9)	438 (54.7)
Nonbinary/transgender	284 (2.5)	136 (2.8)	98 (3.1)	30 (1.1)	20 (2.5)

*Race/ethnicity*					
Hispanic	2,007 (17.5)	821 (16.9)	401 (12.6)	584 (22.2)	201 (25.1)
Black non-Hispanic	1,359 (11.8)	496 (10.2)	211 (6.6)	528 (20.1)	124 (15.5)
Asian\Pacific Islander	781 (6.8)	357 (7.4)	239 (7.5)	117 (4.5)	68 (8.5)
White non-Hispanic	6,915 (60.3)	3,013 (62.2)	2,252 (70.5)	1,273 (48.4)	377 (47.1)
Other	407 (3.5)	159 (3.3)	91 (2.8)	126 (4.8)	31 (3.9)

*Income*					
<$50,000	4,724 (41.2)	1,866 (38.5)	1,005 (31.5)	1,474 (56.1)	379 (47.3)
$50,000 to $99,999	3,489 (30.4)	1,489 (30.7)	1,033 (32.3)	735 (28.0)	232 (29.0)
$100,000 or more	2,913 (25.4)	1,353 (27.9)	1,071 (33.5)	331 (12.6)	158 (19.7)
Unknown	343 (3.0)	138 (2.8)	85 (2.7)	88 (3.3)	32 (4.0)

*Education*					
<High school	223 (1.9)	70 (1.4)	23 (0.7)	110 (4.2)	20 (2.5)
High school graduate	1,362 (11.9)	472 (9.7)	183 (5.7)	601 (22.9)	106 (13.2)
Some college	3,226 (28.1)	1,275 (26.3)	656 (20.5)	1,020 (38.8)	275 (34.3)
College graduate	6,658 (58.1)	3,029 (62.5)	2,332 (73.0)	897 (34.1)	400 (49.9)

*Employment status*⁣^∗^					
Employed	7,406 (64.6)	3,175 (65.5)	2,119 (66.3)	1,596 (60.7)	516 (64.4)
Out of work	1,384 (12.1)	547 (11.3)	313 (9.8)	403 (15.3)	121 (15.1)
Other/unknown	2,679 (23.4)	1,124 (23.2)	762 (23.9)	629 (23.9)	164 (20.5)

*Any *children < 18*in household*					
No	7,769 (67.7)	3,409 (70.3)	2,521 (78.9)	1,368 (52.1)	471 (58.8)
Yes	3,700 (32.3)	1,437 (29.7)	673 (21.1)	1,260 (47.9)	330 (41.2)

*Recent housing insecurity*⁣^∗^					
Usually/always	1,890 (16.5)	759 (15.7)	293 (9.2)	664 (25.3)	174 (21.7)
Rarely/sometimes	3,648 (31.8)	1,574 (32.5)	861 (27.0)	921 (35.0)	292 (36.5)
Never	5,931 (51.7)	2,513 (51.9)	2,040 (63.9)	1,043 (39.7)	335 (41.8)

*Recent food insecurity*⁣^∗^					
No	8,341 (72.7)	3,623 (74.8)	2,696 (84.4)	1,529 (58.2)	493 (61.5)
Yes	3,128 (27.3)	1,223 (25.2)	498 (15.6)	1,099 (41.8)	308 (38.5)

*Potential SARS-CoV-2 exposure risk*					
Less exposure	7,126 (62.1)	3,100 (64.0)	2,197 (68.8)	1,368 (52.1)	461 (57.6)
More exposure	4,343 (37.9)	1,746 (36.0)	997 (31.2)	1,260 (47.9)	340 (42.4)

*Susceptibility to severe COVID-19 disease*					
Less susceptible	9,037 (78.8)	3,834 (79.1)	2,466 (77.2)	2,073 (78.9)	664 (82.9)
More susceptible	2,432 (21.2)	1,012 (20.9)	728 (22.8)	555 (21.1)	137 (17.1)

*Healthcare access*					
Fewer barriers to access	6,518 (56.8)	2,860 (59.0)	2,165 (67.8)	1,124 (42.8)	369 (46.1)
More barriers to access	4,951 (43.2)	1,986 (41.0)	1,029 (32.2)	1,504 (57.2)	432 (53.9)

*Anxiety symptoms*⁣^∗^					
None/mild	8,574 (74.8)	3,554 (73.3)	2,538 (79.5)	1,889 (71.9)	593 (74.0)
Moderate/severe	2,895 (25.2)	1,292 (26.7)	656 (20.5)	739 (28.1)	208 (26.0)
Mean (STD)	6.39 (5.72)	6.69 (5.64)	5.87 (5.25)	6.46 (6.29)	6.35 (5.91)
Median (IQR)	5 (2, 2)	6 (2, 2)	5 (2, 2)	5 (1, 1)	5 (1, 1)

*Depressive symptoms*⁣^∗^					
None/mild	8,403 (73.3)	3,521 (72.7)	2,464 (77.1)	1,839 (70.0)	579 (72.3)
Moderate/severe	3,066 (26.7)	1,325 (27.3)	730 (22.9)	789 (30.0)	222 (27.7)
Mean (STD)	6.63 (6.16)	6.80 (6.03)	6.13 (5.71)	6.90 (6.77)	6.77 (6.52)
Median (IQR)	5 (1, 1)	5 (2, 2)	5 (2, 2)	5 (1, 1)	5 (1, 1)

⁣^∗^Measured at the survey closest to vaccine status. All other variables are measured at study enrollment.

**Table 2:** Impact of COVID-19 vaccination on moderate to severe symptoms of anxiety and depression prior to and during universal availability of the COVID-19 vaccine—CHASING COVID Cohort, USA, December 2020-January 2022.

Vaccination status and vaccine era	Person-trial denominator	Moderate or severe symptoms of anxiety				Moderate or severe symptoms of depression			
		*N*	Prevalence (95% CI)	OR (95% CI)	aOR (95% CI)⁣^∗^	*N*	Prevalence (95% CI)	OR (95% CI)	aOR (95% CI)⁣^∗∗^
*Preuniversal availability*									
Not vaccinated	4846	1171	24.2 (23.0, 25.4)	1.00	1.00	1231	25.4 (24.2, 26.6)	1.00	1.00
Vaccinated	3194	532	16.7 (15.4, 17.9)	0.70 (0.65, 0.76)	0.79 (0.70, 0.89)	653	20.4 (19.0, 21.8)	0.82 (0.77, 0.88)	0.92 (0.82, 1.03)

*Universal availability*									
Not vaccinated	2628	706	26.9 (25.2, 28.6)	1.00	1.00	782	29.8 (28.0, 31.5)	1.00	1.00
Vaccinated	801	219	27.3 (24.3, 30.4)	1.06 (0.92, 1.22)	1.23 (1.00, 1.50)	234	29.2 (26.1, 32.4)	1.03 (0.89, 1.18)	1.11 (0.91, 1.36)

⁣^∗^Adjusted for baseline access to healthcare, baseline age, anxiety at time 0, baseline education, employment at time 0, gender, housing and food insecurity at time 0, and baseline susceptibility to severe COVID-19. Std errors adjusted for repeat subjects. ⁣^∗∗^Adjusted for baseline access to healthcare, baseline age, depression at time 0, baseline education, employment at time 0, gender, housing and food insecurity at time 0, and baseline susceptibility to severe COVID-19. Std errors adjusted for repeat subjects.

**Table 3:** Impact of COVID-19 vaccination on moderate to severe symptoms of anxiety and depression prior to and during universal availability of the COVID-19 vaccine in the subgroup of participants with anxiety or depression symptoms at time 0—CHASING COVID Cohort, USA, December 2020-January 2022.

Vaccination status and vaccine era	Symptoms of anxiety								Symptoms of depression							
	Moderate or severe symptoms					Mean GAD-7 score			Moderate or severe symptoms					Mean PHQ-8 score		
	Person-trial denominator	*N*	Prevalence (95% CI)	OR (95% CI)	aOR (95% CI)⁣^∗^	Mean (STD)	Coeff (95% CI)	Adj coeff (95% CI)⁣^∗^	Person-trial denominator	*N*	Prevalence (95% CI)	OR (95% CI)	aOR (95% CI)⁣^∗∗^	Mean (STD)	Coeff (95% CI)	Adj coeff (95% CI)⁣^∗∗^
*Preuniversal*																
Not vaccinated	1292	840	65.0 (62.4, 67.6)	1.00	1.00	12 (5)			1325	895	67.5 (65.0, 70.1)	1.00	1.00			
Vaccinated	656	361	55.0 (51.2, 58.8)	0.62 (0.53, 0.74)	0.67 (0.57, 0.80)	11 (5)	-1.33 (-1.72, -0.94)	-1.16 (-1.55, -0.76)	730	461	63.2 (59.6, 66.7)	0.79 (0.67, 0.93)	0.83 (0.70, 0.98)	13 (6)	-0.99 (-1.40, -0.59)	-0.82 (-1.23, -0.40)

*Universal*														12 (6)		
Not vaccinated	739	494	66.8 (63.5, 70.2)	1.00	1.00	12 (6)			789	549	69.6 (66.4, 72.8)	1.00	1.00	13 (6)		
Vaccinated	208	142	68.3 (61.9, 74.6)	0.96 (0.73, 1.27)	1.03 (0.78, 1.38)	12 (5)	-0.20 (-0.85, 0.45)	-0.17 (-0.83, 0.50)	222	153	68.9 (62.8, 75.0)	0.93 (0.71, 1.22)	0.94 (0.71, 1.24)	13 (6)	-0.29 (-0.99, 0.42)	-0.15 (-0.86, 0.56)

⁣^∗^Adjusted for baseline access to healthcare, baseline age, baseline education, employment at time 0, gender, housing and food insecurity at time 0, and baseline susceptibility to severe COVID-19. Std errors adjusted for repeat subjects. ⁣^∗∗^Adjusted for baseline access to healthcare, baseline age, baseline education, employment at time 0, gender, housing and food insecurity at time 0, and baseline susceptibility to severe COVID-19. Std errors adjusted for repeat subjects.

## Data Availability

A deidentified dataset is available at https://zenodo.org/record/6127735#.Yi9rnBDML0p2022.

## Appendix

### Construction of Summative Indices

We created 3 summative indices as proxies for potential SARS-CoV-2 exposure, susceptibility to COVID-19 complications, and difficulty with access to healthcare. We drew the indices and assessments from a national survey that explored the experience of adults during the 2009-2010 influenza A (H1N1) pandemic [38, 39]. We used the same exposure and access to care measures as the H1N1 survey and modified the susceptibility index for COVID-19. Each index was a summative score, in which a higher risk response was given a value of 1, and a lower or no risk response was given a value of 0. Therefore, a higher value would indicate more potential SARS-CoV-2 exposure, greater susceptibility, and greater difficulty with access to care and treatment.

First, as a measure of potential SARS-CoV-2 exposure, we included built-environment and work-related items that contributed to the ability to social distance. The built-environment items included living in an urban area, living in a multiunit dwelling (e.g., apartment building), and the ability to avoid public transportation. The work-related items included essential worker status and whether respondents were able to stay home from work or work from home, if needed. As the measure of COVID-19 susceptibility, we used conditions or exposures identified by the Centers for Disease Control and Prevention in March 2020 which increased the risk for COVID-19 complications: age > 60 years, daily smoking, and underlying chronic conditions (chronic lung disease, including chronic obstructive pulmonary disease, emphysema, and chronic bronchitis; serious heart conditions, including angina/coronary heart disease, high blood pressure, and history of myocardial infarction; current asthma; type 2 diabetes; kidney disease; immunocompromised condition; or HIV positive). As the measure of healthcare access, we used factors that affect medical care access: no primary care doctor, concerns about the costs of healthcare, concerns about seeing a doctor because of immigration status, or no healthcare coverage/insurance. We dichotomized each index as less than or equal to the median value for the cohort: more versus less susceptible to COVID-19 complications and more versus less difficulty with access to care. Further details are available [40].

## Supplementary Materials

Supplementary Materials Sociodemographic characteristics by vaccination status in the pre- and universal-vaccine eras among the subgroups with moderate or severe anxiety or depressive symptoms are reported in Supplemental Table 1. The protocol of the target trial to estimate the effect of vaccination on symptoms of anxiety and depression among all adults and among the subgroup of adults with prevalent symptoms of anxiety or depression is outlined in Supplemental Table 2. The number of participants, vaccinated participants, and outcome events in each “trial” to estimate the intention-to-treat effects of COVID-19 vaccination is provided in Supplemental Table 3.
