# Supplementary material for: The Impact of COVID-19 Vaccination on Symptoms of Anxiety and Depression before and after COVID-19 Vaccines Were Universally Available for Adults in the United States
Source: Depress Anxiety. 2024 May 7;2024:9682710. doi: 10.1155/2024/9682710 (PMC11918965; doi:10.1155/2024/9682710)
Supplement: Supplementary Materials — Sociodemographic characteristics by vaccination status in the pre- and universal-vaccine eras among the subgroups with moderate or severe anxiety or depressive symptoms are reported in Supplemental Table 1. The protocol of the target trial to estimate the effect of vaccination on symptoms of anxiety and depression among all adults and among the subgroup of adults with prevalent symptoms of anxiety or depression is outlined in Supplemental Table 2. The number of participants, vaccinated participants, and outcome events in each “trial” to estimate the intention-to-treat effects of COVID-19 vaccination is provided in Supplemental Table 3. [file 9682710.f1.docx]

**Supplemental Table 1. Sociodemogaphic Characteristics by Vaccination Status in the Pre- and Universal-Vaccine Eras Among the Subgroups with Moderate/Severe Anxiety or Depressive Symptoms, Chasing COVID Cohort, US - December 2020- January 2022**

|  | **Moderate or Severe Anxiety Symptoms Subgroup** | | | | | **Moderate or Severe Depressive Symptoms Subgroup** | | | | |
| --- | --- | --- | --- | --- | --- | --- | --- | --- | --- | --- |
|  |  | **Pre-Universal Vaccine Era (December 2020-April 18, 2021)** | | **Universal Vaccine Era (April 19, 2021-January 11, 2022)** | |  | **Pre-Universal Vaccine Era (December 2020-April 18, 2021)** | | **Universal Vaccine Era (April 19, 2021-January 11, 2022)** | |
|  | **Overall, N (%)** | **Unvaccinated, N (%)** | **Vaccinated, N (%)** | **Unvaccinated, N (%)** | **Vaccinated, N (%)** | **Overall, N (%)** | **Unvaccinated, N (%)** | **Vaccinated, N (%)** | **Unvaccinated, N (%)** | **Vaccinated, N (%)** |
|  | 2,895 (100.0) | 1,292 (100.0) | 656 (100.0) | 739 (100.0) | 208 (100.0) | 3,066 (100.0) | 1,325 (100.0) | 730 (100.0) | 789 (100.0) | 222 (100.0) |
| **Age** |  |  |  |  |  |  |  |  |  |  |
| 18-49 | 2,481 (85.7) | 1,099 (85.1) | 530 (80.8) | 666 (90.1) | 186 (89.4) | 2,559 (83.5) | 1,101 (83.1) | 573 (78.5) | 692 (87.7) | 193 (86.9) |
| 50-59 | 261 (9.0) | 119 (9.2) | 74 (11.3) | 53 (7.2) | 15 (7.2) | 313 (10.2) | 138 (10.4) | 88 (12.1) | 66 (8.4) | 21 (9.5) |
| 60+ | 153 (5.3) | 74 (5.7) | 52 (7.9) | 20 (2.7) | 7 (3.4) | 194 (6.3) | 86 (6.5) | 69 (9.5) | 31 (3.9) | 8 (3.6) |
| **Gender** |  |  |  |  |  |  |  |  |  |  |
| Cisgender Male | 1,011 (34.9) | 476 (36.8) | 269 (41.0) | 189 (25.6) | 77 (37.0) | 1,110 (36.2) | 508 (38.3) | 309 (42.3) | 205 (26.0) | 88 (39.6) |
| Cisgender Female | 1,750 (60.4) | 745 (57.7) | 341 (52.0) | 542 (73.3) | 122 (58.7) | 1,803 (58.8) | 740 (55.8) | 366 (50.1) | 572 (72.5) | 125 (56.3) |
| Non-Binary/Transgender | 134 (4.6) | 71 (5.5) | 46 (7.0) | 8 (1.1) | 9 (4.3) | 153 (5.0) | 77 (5.8) | 55 (7.5) | 12 (1.5) | 9 (4.1) |
| **Race/Ethnicity** |  |  |  |  |  |  |  |  |  |  |
| Hispanic | 584 (20.2) | 259 (20.0) | 105 (16.0) | 170 (23.0) | 50 (24.0) | 615 (20.1) | 275 (20.8) | 112 (15.3) | 172 (21.8) | 56 (25.2) |
| Black Non-Hispanic | 330 (11.4) | 130 (10.1) | 45 (6.9) | 121 (16.4) | 34 (16.3) | 360 (11.7) | 138 (10.4) | 52 (7.1) | 134 (17.0) | 36 (16.2) |
| Asian\Pacific Islander | 190 (6.6) | 93 (7.2) | 53 (8.1) | 28 (3.8) | 16 (7.7) | 188 (6.1) | 90 (6.8) | 55 (7.5) | 27 (3.4) | 16 (7.2) |
| White Non-Hispanic | 1,635 (56.5) | 745 (57.7) | 434 (66.2) | 365 (49.4) | 91 (43.8) | 1,734 (56.6) | 758 (57.2) | 487 (66.7) | 392 (49.7) | 97 (43.7) |
| Other | 156 (5.4) | 65 (5.0) | 19 (2.9) | 55 (7.4) | 17 (8.2) | 169 (5.5) | 64 (4.8) | 24 (3.3) | 64 (8.1) | 17 (7.7) |
| **Income** |  |  |  |  |  |  |  |  |  |  |
| <$50,000 | 1,562 (54.0) | 644 (49.8) | 279 (42.5) | 503 (68.1) | 136 (65.4) | 1,699 (55.4) | 705 (53.2) | 330 (45.2) | 523 (66.3) | 141 (63.5) |
| $50,000 to $99,999 | 719 (24.8) | 329 (25.5) | 191 (29.1) | 156 (21.1) | 43 (20.7) | 767 (25.0) | 331 (25.0) | 212 (29.0) | 173 (21.9) | 51 (23.0) |
| $100,000 or more | 527 (18.2) | 278 (21.5) | 170 (25.9) | 56 (7.6) | 23 (11.1) | 494 (16.1) | 246 (18.6) | 168 (23.0) | 58 (7.4) | 22 (9.9) |
| Unknown | 87 (3.0) | 41 (3.2) | 16 (2.4) | 24 (3.2) | 6 (2.9) | 106 (3.5) | 43 (3.2) | 20 (2.7) | 35 (4.4) | 8 (3.6) |
| **Education** |  |  |  |  |  |  |  |  |  |  |
| <High school | 100 (3.5) | 33 (2.6) | 9 (1.4) | 50 (6.8) | 8 (3.8) | 102 (3.3) | 34 (2.6) | 8 (1.1) | 52 (6.6) | 8 (3.6) |
| High school graduate | 456 (15.8) | 160 (12.4) | 46 (7.0) | 212 (28.7) | 38 (18.3) | 505 (16.5) | 181 (13.7) | 66 (9.0) | 219 (27.8) | 39 (17.6) |
| Some college | 920 (31.8) | 375 (29.0) | 165 (25.2) | 293 (39.6) | 87 (41.8) | 1,024 (33.4) | 427 (32.2) | 184 (25.2) | 320 (40.6) | 93 (41.9) |
| College graduate | 1,419 (49.0) | 724 (56.0) | 436 (66.5) | 184 (24.9) | 75 (36.1) | 1,435 (46.8) | 683 (51.5) | 472 (64.7) | 198 (25.1) | 82 (36.9) |
| **Employment status*** |  |  |  |  |  |  |  |  |  |  |
| Employed | 1,765 (61.0) | 812 (62.8) | 429 (65.4) | 413 (55.9) | 111 (53.4) | 1,796 (58.6) | 787 (59.4) | 461 (63.2) | 432 (54.8) | 116 (52.3) |
| Out of work | 561 (19.4) | 225 (17.4) | 118 (18.0) | 165 (22.3) | 53 (25.5) | 633 (20.6) | 257 (19.4) | 134 (18.4) | 183 (23.2) | 59 (26.6) |
| Other/Unknown | 569 (19.7) | 255 (19.7) | 109 (16.6) | 161 (21.8) | 44 (21.2) | 637 (20.8) | 281 (21.2) | 135 (18.5) | 174 (22.1) | 47 (21.2) |
| **Any Children <18 in Household** |  |  |  |  |  |  |  |  |  |  |
| No | 1,827 (63.1) | 849 (65.7) | 504 (76.8) | 352 (47.6) | 122 (58.7) | 1,958 (63.9) | 869 (65.6) | 575 (78.8) | 380 (48.2) | 134 (60.4) |
| Yes | 1,068 (36.9) | 443 (34.3) | 152 (23.2) | 387 (52.4) | 86 (41.3) | 1,108 (36.1) | 456 (34.4) | 155 (21.2) | 409 (51.8) | 88 (39.6) |
| **Recent housing insecurity*** |  |  |  |  |  |  |  |  |  |  |
| Usually/Always | 1,008 (34.8) | 406 (31.4) | 146 (22.3) | 363 (49.1) | 93 (44.7) | 1,061 (34.6) | 425 (32.1) | 163 (22.3) | 372 (47.1) | 101 (45.5) |
| Rarely/Sometimes | 1,000 (34.5) | 461 (35.7) | 226 (34.5) | 244 (33.0) | 69 (33.2) | 1,048 (34.2) | 472 (35.6) | 250 (34.2) | 250 (31.7) | 76 (34.2) |
| Never | 887 (30.6) | 425 (32.9) | 284 (43.3) | 132 (17.9) | 46 (22.1) | 957 (31.2) | 428 (32.3) | 317 (43.4) | 167 (21.2) | 45 (20.3) |
| **Recent food insecurity*** |  |  |  |  |  |  |  |  |  |  |
| No | 1,485 (51.3) | 732 (56.7) | 444 (67.7) | 239 (32.3) | 70 (33.7) | 1,569 (51.2) | 728 (54.9) | 488 (66.8) | 277 (35.1) | 76 (34.2) |
| Yes | 1,410 (48.7) | 560 (43.3) | 212 (32.3) | 500 (67.7) | 138 (66.3) | 1,497 (48.8) | 597 (45.1) | 242 (33.2) | 512 (64.9) | 146 (65.8) |
| **Potential SARS CoV-2 Exposure Risk** |  |  |  |  |  |  |  |  |  |  |
| Less exposure | 1,525 (52.7) | 713 (55.2) | 389 (59.3) | 322 (43.6) | 101 (48.6) | 1,614 (52.6) | 731 (55.2) | 430 (58.9) | 349 (44.2) | 104 (46.8) |
| More exposure | 1,370 (47.3) | 579 (44.8) | 267 (40.7) | 417 (56.4) | 107 (51.4) | 1,452 (47.4) | 594 (44.8) | 300 (41.1) | 440 (55.8) | 118 (53.2) |
| **Susceptibility to Severe COVID-19 Disease** |  |  |  |  |  |  |  |  |  |  |
| Less susceptible | 2,217 (76.6) | 1,025 (79.3) | 502 (76.5) | 535 (72.4) | 155 (74.5) | 2,314 (75.5) | 1,030 (77.7) | 549 (75.2) | 572 (72.5) | 163 (73.4) |
| More susceptible | 678 (23.4) | 267 (20.7) | 154 (23.5) | 204 (27.6) | 53 (25.5) | 752 (24.5) | 295 (22.3) | 181 (24.8) | 217 (27.5) | 59 (26.6) |
| **Healthcare Access** |  |  |  |  |  |  |  |  |  |  |
| Fewer barriers to access | 1,306 (45.1) | 615 (47.6) | 377 (57.5) | 240 (32.5) | 74 (35.6) | 1,386 (45.2) | 628 (47.4) | 415 (56.8) | 259 (32.8) | 84 (37.8) |
| More barriers to access | 1,589 (54.9) | 677 (52.4) | 279 (42.5) | 499 (67.5) | 134 (64.4) | 1,680 (54.8) | 697 (52.6) | 315 (43.2) | 530 (67.2) | 138 (62.2) |
| **Anxiety Symptoms*** |  |  |  |  |  |  |  |  |  |  |
| None/Mild |  |  |  |  |  | 744 (24.3) | 304 (22.9) | 218 (29.9) | 173 (21.9) | 49 (22.1) |
| Moderate/Severe | 2,895 (100.0) | 1,292 (100.0) | 656 (100.0) | 739 (100.0) | 208 (100.0) | 2,322 (75.7) | 1,021 (77.1) | 512 (70.1) | 616 (78.1) | 173 (77.9) |
| Mean (STD) | 14.67 (3.57) | 14.59 (3.42) | 14.33 (3.61) | 15.08 (3.72) | 14.76 (3.65) | 13.22 (4.82) | 13.25 (4.70) | 12.61 (4.87) | 13.72 (4.91) | 13.32 (4.83) |
| Median (IQR) | 14 (12, 12) | 14 (12, 12) | 13 (11, 11) | 14 (12, 12) | 14 (12, 12) | 13 (10, 10) | 13 (10, 10) | 12 (9, 9) | 14 (10, 10) | 13 (10, 10) |
| **Depressive Symptoms*** |  |  |  |  |  |  |  |  |  |  |
| None/Mild | 573 (19.8) | 271 (21.0) | 144 (22.0) | 123 (16.6) | 35 (16.8) |  |  |  |  |  |
| Moderate/Severe | 2,322 (80.2) | 1,021 (79.0) | 512 (78.0) | 616 (83.4) | 173 (83.2) | 3,066 (100.0) | 1,325 (100.0) | 730 (100.0) | 789 (100.0) | 222 (100.0) |
| Mean (STD) | 14.32 (5.27) | 13.98 (5.14) | 13.92 (5.25) | 15.13 (5.35) | 14.86 (5.46) | 15.25 (4.10) | 15.07 (3.98) | 14.89 (3.94) | 15.75 (4.33) | 15.72 (4.19) |
| Median (IQR) | 14 (10, 10) | 14 (10, 10) | 14 (10, 10) | 15 (11, 11) | 15 (11, 11) | 14 (12, 12) | 14 (12, 12) | 14 (12, 12) | 15 (12, 12) | 15 (12, 12) |

*Measured at the survey closest to vaccine status. All other variables are measured at study enrollment

**Supplemental Table 2. Specification and Emulation of a Target Trial of Vaccine Effects on Symptoms of Anxiety and Depression Using Data from the Chasing COVID Cohort**

| **Protocol** | Target Trial | Cohort Design: Target Trial Emulation |
| --- | --- | --- |
| **Aim** | To estimate the effect of vaccination on short-term symptoms of moderate to severe anxiety or depression before and after COVID-19 vaccines were universally available. | Same. |
| **Eligibility** | 18+ years of age during the study period December 2020-December 2021   No vaccine contraindication or precaution (history of severe allergic reaction, allergy to vaccine component, moderate or severe acute illness with or without fever, history of multisystem inflammatory syndrome in adults) | 18+ years as of recruitment (March-August 2020) and completing an assessment between December 2020-December 2021. We cannot replicate exclusion criteria (e.g., vaccine contraindication) |
| **Treatment Strategies** | Vaccination (first shot in series - with expectation that someone would complete the primary series) at baseline   No vaccination at baseline | Same. |
| **Treatment assignment** | Individuals are randomly assigned to a strategy at baseline and study is not blinded to investigators or enrollees | We classified individuals according to the strategy that their data were compatible with at baseline (vaccination or no vaccination). Randomization is emulated via adjustment for baseline covariates: age, gender, education, employment, access to healthcare, housing and food insecurity, susceptibility to severe COVID-19 outcome, and anxiety symptoms for the anxiety model. For the depression model, we adjusted for the same baseline covariates and baseline depression status, excluding baseline anxiety. |
| **Follow-up** | For each eligible individual, follow-up begins at the date of treatment assignment and continues for three months | Follow-up extends to the subsequent assessment, approximately two to three months. |
| **Primary Outcome** | Symptoms of generalized anxiety using the Generalized Anxiety Disorder 7-item (GAD-7) and depression using the Patient Health Questionnaire 8-item (PHQ-8). We dichotomized as symptoms of moderate to severe anxiety (GAD-7 score >10) or depression (PHQ-8 >10). | Same. |
| **Causal contrast** | Intention to treat effect, effect of being assigned to vaccination versus no vaccination before and after vaccines were universally available. | Same (observational analog of ITT). |
| **Statistical analysis** | Intention-to-treat analysis. 1) The comparison of moderate-to-severe anxiety under each treatment strategy stratified by the universal vaccine era. 2) The comparison of moderate-to-severe depression under each treatment strategy stratified by the universal vaccine era. | Same. Further adjustment for baseline covariates. |
| **Design** | Randomized trial. | Cohort conceptualized as a sequence of non-randomized ‘trials’ where participants can enter the next ‘trial’ at each subsequent assessment. In each ‘trial’, we used the corresponding questionnaire information to apply eligibility and identify those newly vaccinated or not newly vaccinated. |

**Supplemental Table 3. Number of participants, vaccinated participants, and outcome events in each “trial’ to estimate intention-to-treat effects of COVID-19 vaccination**

|  |  |  | **Moderate to Severe Anxiety Symptoms** | | **Moderate to Severe Depressive Symptoms** | |
| --- | --- | --- | --- | --- | --- | --- |
|  | **Total** | **Vaccinated** | **Among all** | **Among Vaccinated** | **Among all** | **Among Vaccinated** |
| Trial 1. Dec 16-28, 2020 | 4613 | 143 | 1091 | 41 | 1159 | 47 |
| Trial 2. Feb 10-May 6, 2021 | 4495 | 3228 | 901 | 536 | 1037 | 650 |
| Trial 3. May 25-July 9, 2021 | 1126 | 364 | 309 | 103 | 341 | 111 |
| Trial 4. Sept 23-Oct 21, 2021 | 710 | 198 | 193 | 50 | 215 | 57 |
| Trial 5. Dec 20-Jan 11, 2022 | 525 | 62 | 134 | 21 | 148 | 22 |
